# Histone Deacetylases Regulate Gonadotropin-Releasing Hormone I Gene Expression via Modulating Otx2-Driven Transcriptional Activity

**DOI:** 10.1371/journal.pone.0039770

**Published:** 2012-06-25

**Authors:** Lu Gan, Pei-Yan Ni, Yan Ge, Yun-Fei Xiao, Chang-Yan Sun, Lin Deng, Wei Zhang, Si-Si Wu, Ying Liu, Wei Jiang, Hong-Bo Xin

**Affiliations:** 1 Laboratory of Cardiovascular Diseases and Laboratory of Cellular and Molecular Biology, State Key Laboratory of Biotherapy, West China Hospital, Sichuan University, Chengdu, People's Republic of China; 2 Institute of Translational Medicine, Nanchang University, Nanchang, People's Republic of China; Kaohsiung Chang Gung Memorial Hospital, Taiwan

## Abstract

**Background:**

Precise coordination of the hypothalamic-pituitary-gonadal axis orchestrates the normal reproductive function. As a central regulator, the appropriate synthesis and secretion of gonadotropin-releasing hormone I (GnRH-I) from the hypothalamus is essential for the coordination. Recently, emerging evidence indicates that histone deacetylases (HDACs) play an important role in maintaining normal reproductive function. In this study, we identify the potential effects of HDACs on *Gnrh1* gene transcription.

**Methodology/Principal Findings:**

Inhibition of HDACs activities by trichostatin A (TSA) and valproic acid (VPA) promptly and dramatically repressed transcription of *Gnrh1* gene in the mouse immortalized mature GnRH neuronal cells GT1–7. The suppression was connected with a specific region of *Gnrh1* gene promoter, which contains two consensus Otx2 binding sites. Otx2 has been known to activate the basal and also enhancer-driven transcription of *Gnrh1* gene. The transcriptional activity of Otx2 is negatively modulated by Grg4, a member of the Groucho-related-gene (Grg) family. In the present study, the expression of Otx2 was downregulated by TSA and VPA in GT1–7 cells, accompanied with the opposite changes of *Grg4* expression. Chromatin immunoprecipitation and electrophoretic mobility shift assays demonstrated that the DNA-binding activity of Otx2 to *Gnrh1* gene was suppressed by TSA and VPA. Overexpression of Otx2 partly abolished the TSA- and VPA-induced downregulation of *Gnrh1* gene expression.

**Conclusions/Significance:**

Our data indicate that HDAC inhibitors downregulate *Gnrh1* gene expression via repressing Otx2-driven transcriptional activity. This study should provide an insight for our understanding on the effects of HDACs in the reproductive system and suggests that HDACs could be potential novel targets for the therapy of GnRH-related diseases.

## Introduction

Normal reproductive function requires the precise orchestration and integration of sex steroids secretion to effectively coordinate the hypothalamic-pituitary-gonadal axis [1]. As the central regulator, gonadotropin-releasing hormone I (GnRH-I) is pulsatily secreted from some highly restricted, yet scattered particular nuclei within the hypothalamus, and controls the synthesis and release of luteinizing hormone (LH) and follicle-stimulating hormone (FSH) in pituitary [2], [3]. Dysfunction or hyperfunction of GnRH neurons leads to various pathophysiologic disorders, including infertility [4], hypogonadotropic hypogonadism [5], hypothalamic amenorrhea [6] and central precocious puberty [7]. Despite GnRH agonists/analogues and GnRH antagonists are widely applied in clinic, these compounds still remain skepticism for some unsolved issues, including pharmacokinetic, safety and commercial profiles [8]. Therefore, elucidation of the exact molecular mechanisms controlling *Gnrh1* gene expression will improve our understanding on abnormal gonadotropin secretion in various GnRH-related disorders and provide new strategies for treatment of these diseases.

Histone deacetylases (HDACs) have gained an increasing attention for their crucial roles in numerous physiological and pathological processes via dynamically regulating gene expression. According to the profiles of global gene expression, a range of 2–20% of genes in the genome is affected by HDAC inhibitors (HDACIs) [9]–[11], indicating that a highly restricted set of cellular genes is sensitive to changes in histone acetylation [12]. Recently, emerging evidence indicates the involvement of HDACs in maintaining normal reproductive function. HDACs participate in spermatogenesis [13], mediate *Lhβ* and *Fshβ* gene repression in immature gonadotropes [14], [15] and inhibit androgen receptor transcriptional activity [16]. However, the exact roles of HDACs in the modulation of neuronal function of GnRH neurons have remained unclear and need to be further delineated.

So far, at least 18 HDACs have been identified in mammals, in which they are divided into four classes based on their structure and functions. Both Class I (HDACs 1–3 and 8) and class II (HDACs 4–7 and 9–10) HDACs are Zn-dependent enzymes which usually combine with other proteins to form a large multi-protein complex that increases the stringency of HDACs recruitment to a particular locus in chromatin to deacetylate histones [14], [17], [18]. In the present study, we initially observed that *Hdac1–10* genes were co-expressed in the mouse immortalized GnRH neuronal cells GT1–7, which are representative of mature postmigratory GnRH neurons. Two HDAC inhibitors (HDACIs), trichostatin A (TSA) and valproic acid (VPA), induced marked inhibition of *Gnrh1* gene transcription, and the suppression was connected with a specific region of *Gnrh1* gene promoter, which contains two consensus Otx2 binding sites. Otx2, a vertebrate homologue of *Drosophila* orthodenticle, has been demonstrated to activate basal and also enhancer-driven transcription of *Gnrh1* gene in several vertebrate species [19], [20]. We hypothesized that Otx2 may be involved in the HDACIs-induced attenuation of *Gnrh1* transcription. The analysis of transcript and protein levels showed that *Otx2* expression was simultaneously inhibited by HDACIs treatments. Chromatin immunoprecipitation (ChIP) and electrophoretic mobility shift assays (EMSA) demonstrated that TSA and VPA induced the suppression of the DNA-binding activity of Otx2 to *Gnrh1* gene. Overexpression of Otx2 partly abolished the TSA- and VPA-induced downregulation of *Gnrh1* gene expression. In addition, Grg4 (Groucho-related-gene 4), a co-repressor regulating *Gnrh1* transcription, repressed the Otx2-directed activation of *Gnrh1* transcription by direct interaction with Otx2. The increases in the expression of *Grg4* and accumulation of Grg4 protein in nuclei followed by HDACIs treatments also supported our conclusion that HDACs regulate the *Gnrh1* gene transcription partly via modulating Otx2-driven transcriptional activity.

## Results

### TSA and VPA significantly decreased the synthesis and releases of GnRH-I in GT1–7 cells

**Figure 1:**
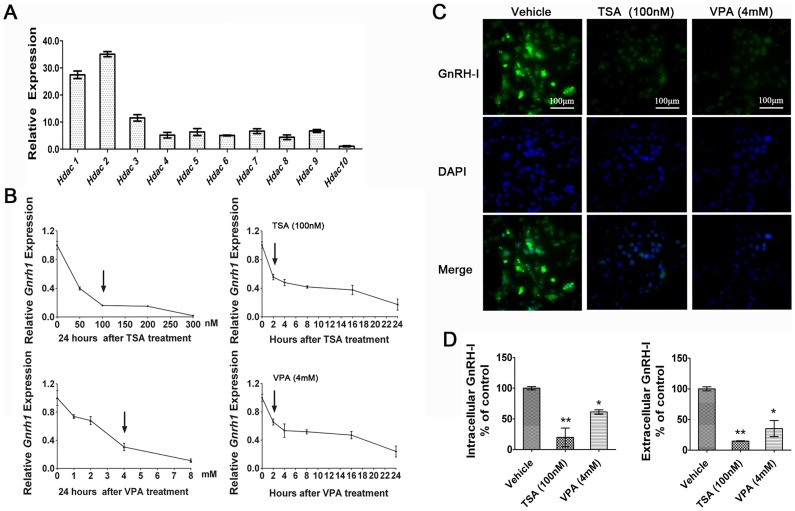
HDACIs-induced repression of *Gnrh1* gene expression in GT1–7 cells. (A) Expressions of endogenous *Hdacs* gene in GT1–7 cells were determined by real-time PCR analysis. The value for the lower expression of *Hdac10* was set as 1.0. (B) The *Gnrh1* transcripts were quantified in GT1–7 cells exposed to various concentrations of TSA or VPA with different times. TSA and VPA induced *Gnrh1* transcripts in time- and dose-dependent manners. The *Gnrh1* mRNA in GT1–7 cells was significantly downregulated by 100 nM TSA and 4 mM VPA within 2 hours. Values for control samples were set as 1.0. (C) Immunofluorescence staining of GnRH-I pre-peptide in GT1–7 cells was performed after treatments of TSA and VPA for 24 hours. All the cultured cells were stained with FLuro 488 for the GnRH-I pre-peptide (green) or with DAPI for nuclei (blue). (D) GnRH-I pre-peptide in the cells and GnRH-I mature peptide in cell culture media were measured by ELISA assay. Values for cells treated with vehicle were set as 100%. Bars represent ± SD from three independent experiments. (n = 3; *p<0.05, ** *p*<0.01; One-way ANOVA).

The endogenous *Hdac* transcripts were detected in GT1–7 cells by real-time PCR analysis, showing that *Hdac1–10* genes were co-expressed with *Hdac1* and *Hdac2* being the predominant transcripts (Figure 1A). These results provided a basic evidence for the potential roles of HDACs in regulating *Gnrh1* expression.

The *Gnrh1* transcripts in GT1–7 cells were noticeably repressed in dose- and time-dependent manners following HDACIs treatments (Figure 1B). When the cells were exposed to 100 nM TSA and 4 mM VPA, the *Gnrh1* transcripts decreased 44.6% and 35.5% within 2 hours, and markedly decreased 84.1% and 76.3% 24 h after HDACIs treatments, respectively. Moreover, the results were confirmed by the immunofluorescence staining (Figure 1C) and ELISA assay (Figure 1D), showing that HDACIs induced significant decreases in the concentrations of intracellular GnRH-I pre-peptides in GT1–7 cells and secretions of GnRH-I mature peptides in cell culture media 24 h after co-incubation with HDACIs.

### TSA and VPA repressed *Gnrh1* gene transcription

**Figure 2:**
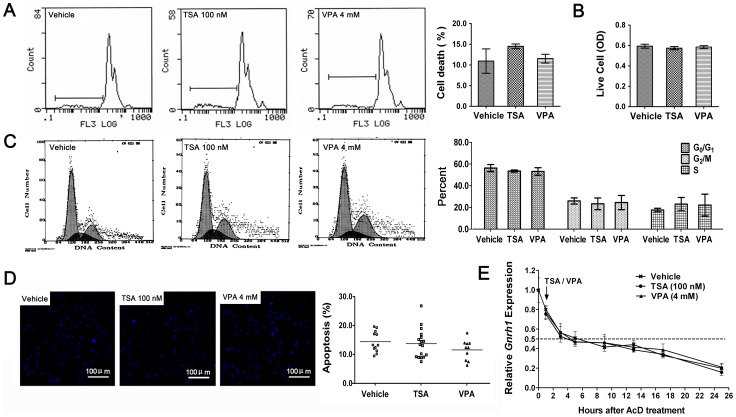
HDACIs-induced inhibiton of *Gnrh1* gene transcription. (A–D) Effects of HDACIs on cytotoxicity, cell cycle progression and apoptosis. (A) Cytotoxicities of HDACIs were detected by flow cytometric (n = 3) and (B) the survival rates of the cells were detected by MTT assay (n = 10). (C) The effects of HDACIs on cell cycle progression were analyzed by PI flow cytometry assays (n = 3). There is no difference among the groups (One-way ANOVA). (D) The apoptosis of the GT1–7 cells treated with HDACIs for 24 h was detected by Hoechst 33258 fluorescent staining. The number of cells present in each image was 50–100. The percentage of apoptotic cells was calculated by dividing the numbers of cells with bright and small nuclei with the total numbers of cells in the image. There is no difference among the groups (n>10, One-way ANOVA). (E) HDACIs inhibited *Gnrh1* gene transcription rather than decreased the stability of *Gnrh1* mRNA. GT1–7 cells were pretreated with 0.01 µM AcD for 1 hour prior to the addition of HDACIs. The arrow represents the point of time for adding HDACIs. The mRNA level was examined by real-time RT-PCR. The value for sample treated by AcD at 0 h was used as control and set as 1.0. (n = 3).

Cell cycle arrest and cytotoxicity have been known to repress gene expression. In order to eliminate the cytotoxicity of the drugs, the dying cells and cell viability were detected by flow cytometric analysis (Figure 2A) and MTT assay (Figure 2B), respectively. The cell cycle progression was detected by propidium iodide (PI) staining (Figure 2C). The results showed that the cell cycle progression and viabilities were not affected by treatments with 100 nM TSA or 4 mM VPA for 24 h. Since only dead cells can be detected by PI staining, Hoechst 33258 staining (Figure 2D) was used to enumerate apoptotic nuclei. The data showed that the apoptosis cannot be induced by both of the inhibitors at the given concentrations.

The levels of gene transcripts in the cells were determined by the rates of mRNA synthesis and decay. Actinomycin D (AcD), an RNA polymerase inhibitor, was used to investigate whether HDACIs influence *Gnrh1* gene transcription or mRNA stability. Our results showed that *Gnrh1* transcripts decreased to half at 3 h when only AcD was present (Figure 2E). Pretreatment with AcD for 1 hour almost completely blocked the TSA- and VPA-induced decreases in *Gnrh1* transcripts, suggesting that TSA and VPA repressed *Ghrh1* gene transcription individually rather than affected mRNA stability.

### HDACIs-induced suppression of *Gnrh1* gene transcription was connected to a specific region of *Gnrh1* gene promoter

**Figure 3:**
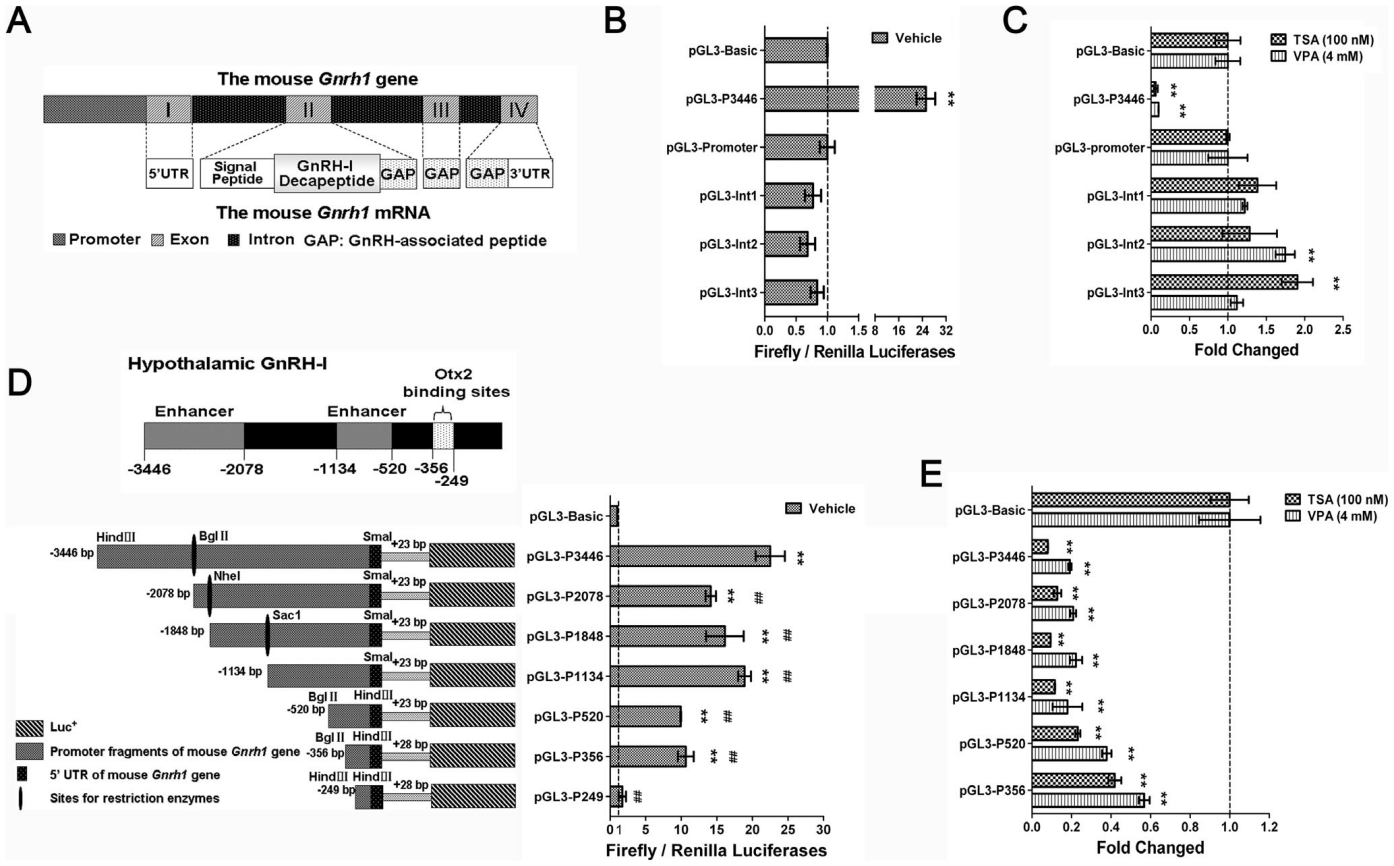
HDACIs-induced inhibition of *Gnrh1* gene transcription associated with a specific region of *Gnrh1* gene promoter. (A) Schematic representation of the parts of the mouse *Gnrh1* gene and mRNA [3]. The effects of promoter (B) and introns (D) on basal *Gnrh1* expression were evaluated by the values of firefly/renilla luciferase activities. The insets in (D) show schematic diagram about constructions of reporter plasmids used in promoter segment deletion analysis (Left) and the elements contribute to the hypothalamus-specific expression of *Gnrh1* (above). The changes of luciferase activities mediated by TSA (C) and VPA (E). The changed folds were calculated by dividing the value of Firefly/Renilla luciferase activity in each group exposed to HDACIs with the value obtained from the group transfected with the same reporter constructs exposed to vehicle. Values for empty vector transfected groups were used as controls and were set as 1.0. (n = 3; *p<0.05, ** *p*<0.01; One-way ANOVA).

The mouse *Gnrh1* gene contains four exons and three introns, and simultaneously encodes the GnRH-I decapeptide and the initial amino acids of GnRH-associated peptide (GAP) [3], [21], [22] (Figure 3A). The neuron-specific promoter in the mouse *Gnrh1* gene is located between −3446 bp to +28 bp [20], [23]. We generated a series of deletion mutants of the promoter as well as introns 1–3 of the mouse *Gnrh1* gene in the pGL3 reporter system to identify the corresponsive region (Figure 3B & D). As shown in Figure 3C, TSA and VPA noticeably inhibited the *Gnrh1* promoter activity by 91.2% and 80.5%, respectively, after 24 h treatments. Unexpectedly, TSA and VPA upregulated the reporter gene activities by 90.4% in intron 3 and 74.6% in intron 2, respectively, suggesting some potential regulatory elements in introns 2 and 3 mediated the enhancement of *Gnrh1* expression in the presence of HDACIs (Figure 3C).

In the promoter segment deletion analysis (Figure 3D), when the promoter was deleted to −2087 bp and −520 bp, the luciferase activities in GT1–7 cells were noticeably decreased without HDACIs treatments, suggesting that potential enhancer elements located in the regions (−3446∼−2087 bp and −1134∼−520 bp). When the promoter was deleted to −249 bp, the luciferase activities downstream were near the value in control group, indicating that the 107-base pair fragment (−356∼−249 bp) is the minimum promoter mediating *Gnrh1* expression in GnRH neurons. The result was consistent with the results of Kim's report, and the 107 bp fragment contains two Otx2 binding sites [20]. As shown in Figure 3E, in the deletion mutant up to −1334 bp, the luciferase activities were dramatically inhibited by TSA and VPA (88.4% and 82.1%, respectively). But when deleted to −356 bp, the luciferase activities gradually recovered to 41.9% and 56.8%, respectively. The data suggested that the region between −1134 and +23 bp mediated the repression, and approximate half of the inhibition was mediated by the region (−356∼+23 bp). A similar tendency in the change of luciferase activities was observed in GT1–7 cells exposed to VPA (Figure 3E), suggesting that the class I HDACs regulate *Gnrh1* expression through the *Gnrh1* promoter region.

### TSA and VPA induced acetylation of histone H3 in the specific region of *Gnrh1* promoter

**Figure 4:**
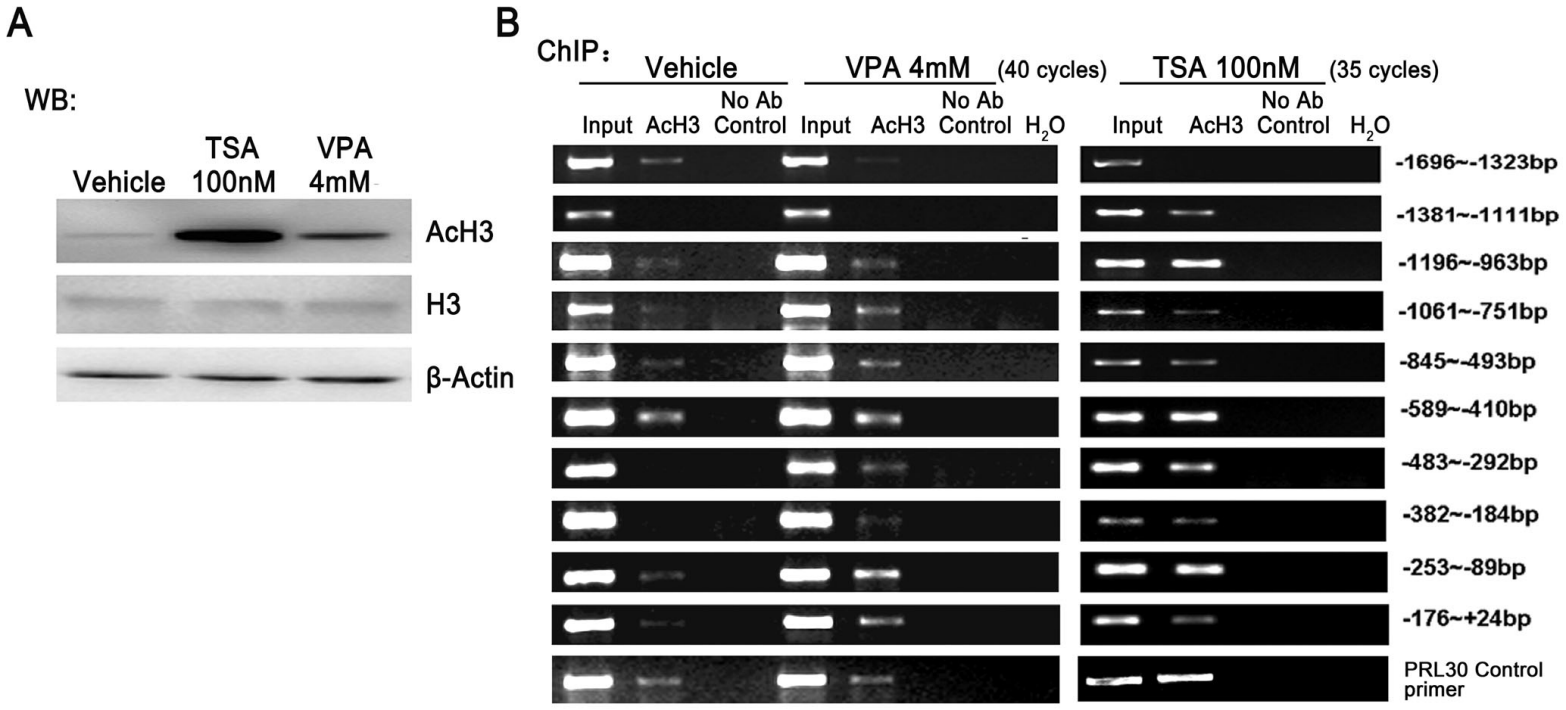
Determination of acetylation level of histone H3 in the promoter of *Gnrh1* gene induced by TSA or VPA. (A) The TSA or VPA-induced acetylation of histone H3 was confirmed by western blot analysis with an anti-acetylated histone H3 (AcH3) and histone H3 antibody. (B) The degree of acetylation of histone H3 in *Gnrh1* promoter was identified by ChIP analysis. Immunoprecipitation was performed with an anti-AcH3 antibody.

Hyperacetylation of histones could open the compacted chromatin to induce transcription activation. Defined by DNase I sensitivity and histone H3 acetylation and methylation assays, Larder and his colleagues show that the activation of *Gnrh1* transcription in GT1–7 cells is due to the active chromatin statue of the regulatory region in promoter [24]. Therefore, the acetylation of histone H3 was detected in the cells following HDACIs treatments. Western blot analysis showed that acetylated histone H3 expression was significantly elevated in GT1–7 cells treated with TSA or VPA (Figure 4A). To characterize such regulatory region with better resolution, ChIP assay was performed to examine the acetylation alteration within the promoter sequence identified by reporter gene assay. The PCR analysis following ChIP showed that acetylation of histone H3 upon TSA and VPA treatments occurred in the regions (−1196∼+24 bp) in *Gnrh1* promoter, respectively (Figure 4B).

### HDACIs inhibited the transcriptional activity of Otx2

**Figure 5:**
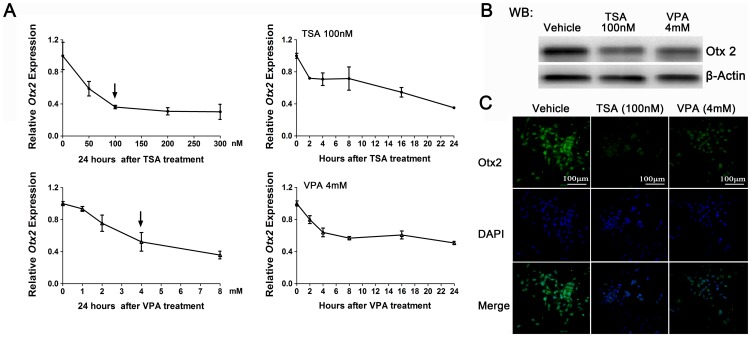
Effects of HDACIs on *Otx2* expression in GT1–7 cells. (A) Expression of *Otx2* mRNA was significantly repressed by TSA (left) and VPA (right) treatments in dose and time-dependent manners using real-time PCR analysis in GT1–7 cells. Values for control samples were set as 1.0. (n = 3). (B) and (C) The protein levels of Otx2 in GT1–7 cells 24 h after HDACIs treatments were determined by western blot (B) and immunofluorescence staining (C), respectively. All the cultured cells were stained with FLuro 488 for the Otx2 protein (green) or with DAPI for nuclei (blue).

Otx2 is critical for basal and neuronal specific expression of *Gnrh1* [20]. In the presence of TSA and VPA, *Otx2* transcripts were dramatically repressed in GT1–7 cells in dose- and time-dependent manners (Figure 5A). When the cells were exposed to 100 nM TSA or 4 mM VPA, *Otx2* transcripts reduced 28.0% and 20.1% within 2 hours, and significantly decreased 64.9% and 49.3% 24 h after HDACIs treatments, respectively. The Otx2 protein levels were also obviously reduced in GT1–7 cells showed by western blot analysis (Figure 5B) and immunofluorescence staining (Figure 5C).

**Figure 6:**
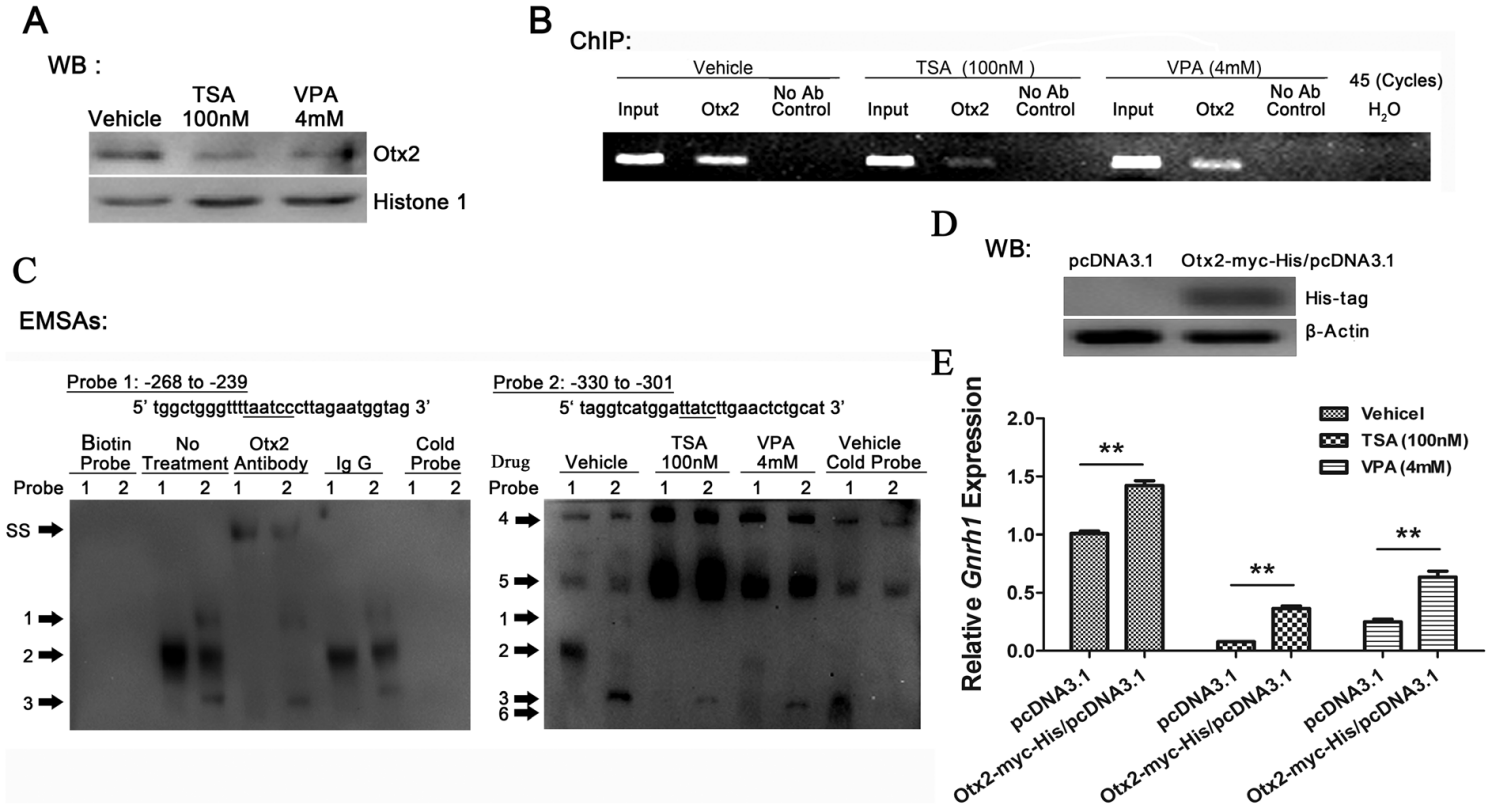
Otx2-mediated transcription activation of *Gnrh1* gene modulated by HDACs. (A) Accumulation of Otx2 in nuclei was repressed by HDACIs in GT1–7 cells. The affinity of Otx2 to the proximal *Gnrh1* gene promoter was suppressed by HDACIs determined by ChIP (B) and EMSA analysis (C). EMSA was performed with GT1–7 nuclear protein extracts using the DNA probes containing the Otx2-binding sites in the mouse *Gnrh1* promoter. A supershifted protein complex (*SS*) was observed on both probes after addition of an Otx2 antibody. A 200-fold excess of unlabeled probes didn't eliminate binding of Otx2 to both probes (complex 2). Complex 1, 3 and 4–6 might non-specific binding. (D) Western blot analysis of protein extracts obtained from GT1–7 cells transfected with pcDNA™ 3.1/*myc*-His C empty vector or Otx2-myc-His/pcDNA3.1 expression vector. (E) HDACIs-induced dowenregulation of *Gnrh1* mRNA was partly rescued by overexpression of Otx2. The value for empty vector transfected control group without drug treatment was set as 1.0. (n = 3; *p<0.05, ** *p*<0.01; Unpaired *t*-test).

When co-incubated with 100 nM TSA or 4 mM VPA for 24 h, the concentrations of Otx2 proteins in cells nuclei were obviously reduced (Figure 6A). The results of ChIP assay showed that the binding ability of Otx2 to neuron-specific elements (−356∼−249 bp) in *Gnrh1* promoter obviously decreased after HDACIs treatments (Figure 6B). To further analyze the affinities of Otx2 to the two conserved binding sites in mouse *Gnrh1* promoter respectively, EMSA was performed with probes containing the −268∼−239 bp and −330∼−301 bp sequences [20] (Figure 6C, above). In GT1–7 cells, Otx2 bound to both the probes (Figure 6C, complex 2) and the Otx2 antibody caused a supershift of this complex (Figure 6C, complex SS). Unexceptly, the complexes 4 and 5 did not disappear in competition with excess unlabeled probes, and the densities of the complexes were significantly increased in the presence of HDACIs (Figure 6C), suggesting that certain proteins or complexes bind to the regions of *Gnrh1* promoter surrounding the confirmed Otx2 consensus sites and be involved in the HDACIs-induced attenuation of *Gnrh1* transcription.

Otx2 overexpression was induced by Otx2-myc-His/pcDNA3.1 transfected in GT1–7 cells (Figure 6D), which could partly rescue the suppression of *Gnrh1* mRNA induced by HDACIs (Figure 6E). All the results showed that the downregulation of *Gnrh1* expression was paralleled with the decrease in *Otx2* expression and repression of its function.

**Figure 7:**
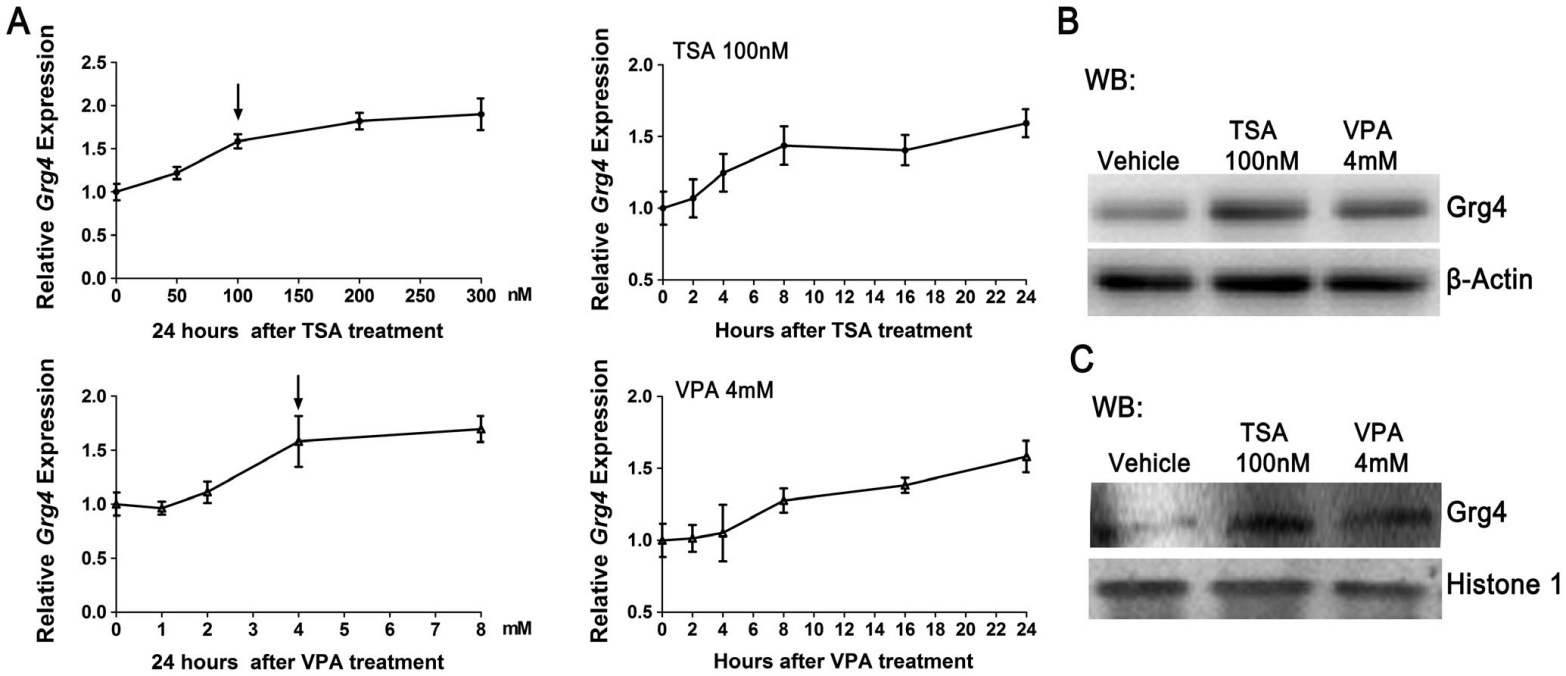
Effects of HDACIs on Grg4 co-repressor in GT1–7 cells. (A) G*rg4* transcripts were significantly elevated following TSA (left) and VPA (right) treatments in dose and time-dependent manners using real-time PCR analysis in GT1–7 cells. Values for control samples were set as 1.0. (n = 3). (B) The total protein levels and (C) nuclear protein levels of Grg4 in GT1–7 cells 24 h after HDACIs treatments were determined by western blot.

Groucho-related-gene 4 (Grg4) has been demonstrated to directly interact with Otx2 to repress Otx2-directed activation of *Gnrh1* transcription in GT1–7 cells [24]. In this study, the results showed that the expression of *Grg4* was elevated in dose- and time-dependent manners in the presence of HDACIs (Figure 7A). Twenty-four hours after incubation with 100 nM TSA or 4 mM VPA, *Grg4* transcripts were increased by 58.6% and 57.2%, respectively. The Grg4 protein levels (Figure 7B), especially the concentrations of Grg4 proteins in cell nuclei (Figure 7C) were obviously upregulated in GT1–7 cells 24 h after HDACIs treatments, indicating that the Grg4-mediated repression was enhanced.

## Discussion

Conceptually, hyperacetylation of lysines in the core histones would enhance gene expression via opening the chromatin structure and easily recruiting transcription-associated proteins to the DNA template. Thus, it is counterintuitive that HDACIs would repress a gene expression rather than activate it. However, a small number of genes, including *p16* [11], *Bcl-2* [25], *Vegf* [26], [27] and *Wt1* [12], have been shown to be downregulated by HDACIs. Here, we report that *Gnrh1* belongs to the class of genes that are downregulated by the TSA and VPA. TSA, a pan-HDAC inhibitor, inhibits both class I and II HDACs. VPA, a potent anti-convulsant and a short-chain fatty acid, acts as a selective class I HDAC inhibitor that especially inhibits HDACs 1, 2, 3 and 8 in a millimolar range without influencing the activities of HDACs 6, 7 and 9 [18]. Thus, our results indicate that at least class I HDACs regulated *Gnrh1* expression. Based on the rapid downregulation of *Gnrh1* transcripts within 2 hours, we could presume that the suppression of *Gnrh1* expression is an early event following HDACIs treatments.

There may be two possibilities for the HDACIs-induced repression of *Gnrh1* expression: directly downregulating the *Gnrh1* transcription, or influencing mRNA stability to promote mRNA degradation. To rule out the second possibility, the RNA polymerase inhibitor AcD is used to inhibit gene transcription. In our study, the results show that the half-life of *Gnrh1* mRNA may be about 3 hours. TSA and VPA do not induce *Gnrh1* mRNA degradation and *Gnrh1* mRNA downregulation required ongoing RNA synthesis.

The elements in the promoter driving tissue-specific expression of mouse *Gnrh1* have well been characterized [3], [28]. A distal enhancer (−3446∼−2087 bp) and the proximal promoter (−346∼+28 bp) have been identified as crucial regions for hypothalamic *Gnrh1* expression [20], [23], which is confirmed by the promoter segment deletion analysis. In the present study, we have demonstrated that HDACIs-induced inhibition of *Gnrh1* transcription in GT1–7 cells is associated with a specific region (−1134∼+23 bp) in *Gnrh1* gene promoter and the region between −356 bp and +23 bp mediates a half of suppression.

It has been demonstrated that the *Gnrh1* transcription is associated with the chromatin status of the regulatory region in *Gnrh1* promoter. Larder and colleague believed that compared to the non-neuronal NIH3T3 cells and immature GnRH neuronal cells (GN11), the acetylation of histone H3 in *Gnrh1* promoter in GT1–7 is responsible for the activation of *Gnrh1* transcription via remodeling the chromatin and recruiting transcription factors [24]. In the present study, the acetylation of histone H3 significantly has been shown to be increased in GT1–7 cells incubated with TSA or VPA. ChIP assay show that histone H3 in the region between −1196 and +24 bp in *Gnrh1* promoter is highly acetylated in the presence of TSA or VPA. Thus, it is counterintuitive that the expression of *Gnrh1* is inhibited by HDACIs via the direct effects of HDACs on *Gnrh1* promoter. In the previous studies, little progress has been achieved in clarifying the exact molecular mechanisms of HDACIs-induced attenuation of gene transcription. Some studies show that increased acetylation of histones in regulatory region would lead nucleosomes move and remodel in specific loci to mask critical cis-acting sites and repress gene expression [12]. The enhancer and silencer regions in genes with different extents of acetylation modified by HDACs may recruit more repressor [12]. Moreover, the HDACs-mediated alternations of transcription factors/repressor expression and function have been emphasized in the modulation of gene transcription [29]. These transcriptional regulatory proteins include p53 [30], p21 [31], NF-κB [32] and E2F1 [33]. Based on the hyperacetylation of histone H3 in *Gnrh1* promoter in the presence of HDACIs, the mechanism mentioned above may be more crucial for the repression.

There are two conserved binding sites for Otx2 in the certain region identified by reporter gene assay [20]. Otx2 increases the basal and enhancer-driven *Gnrh1* gene transcription [19], [20]. During embryogenesis [34] and adulthood [19], [35], Otx2 is colocalized with GnRH-I and regulates the maturation of GnRH neurons and the appropriate synthesis of GnRH-I. Deficiency of Otx2 is responsible for human hypogonadotropic hypogonadism [36]. Recently, Chen and colleagues report that the *Otx2* transcripts in explanted cultures of newborn murine retinae are dramatically repressed by TSA or VPA [37]. In our present study, the *Otx2* transcripts in GT1–7 cells are suppressed by TSA or VPA in dose- and time-dependent manners within 2 hours, which is consistent with the changes of *Gnrh1* expression. Meanwhile, western blot analysis and immunofluorescence staining assay demonstrate that TSA and VPA induce obvious decreases in the intracellular protein level and the nuclear translocation of Otx2. The results from ChIP and EMSA assays show that the affinity of Otx2 to *Gnrh1* gene promoter is significantly inhibited by HDACIs, which may be due to the less *Otx2* expression and less Otx2 protein accumulated in nuclei. The HDACIs-induced the decrease in *Gnrh1* mRNA was partly reversed via overexpression of Otx2. The exact mechanisms of HDACIs-induced downregulation of Otx2 expression have remained unclear and need to be further delineated. Based on the above results, we reasonably conclude that Otx2-driven transcription activation of *Gnrh1* gene is partly modulated by HDACs.

Moreover, emerging evidence has shown that interactions between Otx2 and cofactors regulate Otx2 transcriptional activity [38]–[40]. Grg4, a member of the Groucho-related gene family, has a glycine/prolin-rich region to recruit HDACs. Many studies show that Grg4 switches off transcription via an HDAC-dependent or independent mechanism. Recently, it has been demonstrated that Grg4 represses Otx2-directed activation of *Gnrh1* transcription in GT1–7 cells via physically interacting with Otx2 rather than recruiting HDACs [24]. In our present study, we observe that *Grg4* expression is regulated by HDACs, showing that the expression of *Grg4* is upregulated by TSA and VPA in dose- and time-dependent manners. Inconsistent with rapid attenuation of *Gnrh1* and *Otx2* transcription within 2 hours after HDACIs treatments, the *Grg4* transcripts is slowly elevated, indicating the limited effect of Grg4 on the early inhibition of *Gnrh1* transcription induced by HDACIs. The accumulation of Grg4 protein in nuclei induced by HDACIs may repression the Otx2-induced transcriptional activation, and the results may explain the phenomenon of a part reverse in *Gnrh1* mRNA mediated by exogenous Otx2 with HDACIs treatments. All the results suggest that a potential modulation effect of HDACs on Otx2-induced activation of *Gnrh1* transcription may be via Grg4-dependent repression. In addition, in the present study, EMSA show that the intensities of two top bands are obviously increased following HDACIs treatments, suggesting that the suppression of *Gnrh1* expression might be mediated by proteins or complexes which bind to the regions of *Gnrh1* promoter surrounding the confirmed Otx2 consensus sites. Therefore, further studies should be conducted to identify the potential trans-acting factors which are responsible for the HDACs-induced suppression of *Gnrh1* expression or co-repressor which inhibit Otx2 function. Grg4 may be a candidate of these proteins.

In summary, to our knowledge, the present study provides evidence for the first time that (1) TSA and VPA repress *Gnrh1* gene transcription, (2) the HDACIs-induced repression is related to a specific region of the *Gnrh1* gene promoter (−1134∼+23 bp), which contains two consensus Otx2 binding sites, (3) the Otx2-directed activation of *Gnrh1* gene transcription is modulated by HDACs. This study has complemented the potential roles of HDACs in the regulation of the reproductive system and suggests a potential avenue for the anti-GnRH therapies in the future.

## Materials and Methods

### Chemicals

Trichostatin A (TSA), valproic acid (VPA), and actinomycin D (AcD) were purchased from Sigma Chemical Co. TSA, actinomycin D, cycloheximide were diluted in dimethyl sulfoxide (DMSO), and VPA was diluted with phosphate buffered saline (PBS).

### Cell culture and drug treatment

The mouse immortalized GnRH neuronal cell line GT1–7 [19], [41], [42] (kindly provided by Dr. Pamela Mellon, Salk Institute for Biological Studies, USA) was grown under routine conditions. When the cells reached to 70–80% confluence, they were separately treated with TSA and VPA. In the experiments involving AcD treatments, the cells were pretreated with 0.01 µM AcD for one hour prior to the addition of 100 nM TSA and 4 mM VPA.

### mRNA extraction and RT-PCR

Total RNA was isolated and reverse transcribed to cDNA by M-MLV reverse transcriptase (Invitrogen). The real-time quantitative PCR reactions were carried out with SYBR® Premix Ex Taq™ II real-time PCR kit (Takara, Japan). The primers used are listed in Table S1. The mRNA level of *GAPDH* in each sample was used as an internal control. Relative quantities were analyzed by the 2^−ΔΔCt^ method. In most case, the samples with vehicle treatment were used as controls (set as 1.0). All reactions were carried out in duplicate.

### Enzyme linked immunosorbent assay (ELISA)

The GT1–7 cells were incubated with serum-free DMEM for 24 h to synchronize the cell cycles. Subsequently, twenty four hours after co-incubation, supernatants (medium) and cells were harvested. The cells were lysed in 100 µl PBS containing 0.1% Triton X-100 at 0°C [42]. Supernatants and lysates were assayed for GnRH-I by the Luteinizing Hormone-Releasing Hormone (LH-RH) EIA Kit (Phoenix pharmaceuticals) according to the manufacturer's recommendations.

### Immunofluorescence

Twenty-four hours after HDACIs treatments, the cells were fixed with 4% paraformaldehyde in PBS and permeabilized with 1% Triton X-100 for 30 min, and then blocked with 3% BSA and 1% Triton for 15 min. Following incubation with anti-GnRH-I (1∶20, Santa Cruz) or anti-Otx2 (5 µg/ml, Abcam) for 2 h, the cells were washed twice with PBS and incubated with Alexa Fluor 488-conjugated antibody (1 µg/ml, Invitrogen) for 1.5 h. The nuclei were stained by 4′, 6-diamidino-2-phenylindole (DAPI, 20 µg/ml, Sigma) for 10 min. Images were collected by a digital camera (DXM 1200; Nikon) attached to a microscope.

### Constructs, transfection, and reporter gene assay

The Otx2 cDNA fragment was obtained by RT-PCR and subcloned into the pcDNA™ 3.1/*myc*-His C vector (Invitrogen) to form the plasmid Otx2-myc-His/pcDNA3.1. 3446mGnRH-LUC and 2078mGnRH-LUC plasmids were kindly provided by Dr. Kim (The University of Chicago, USA). The segments of the *Gnrh1* promoter (−3446∼+23 bp, −2078∼+23 bp, −1848∼+23 bp, −1134∼+23 bp) were digested from 3446mGnRH-LUC and subcloned into the pGL3-Basic vector to form the plasmids pGL3-P3446, pGL3-P2087, pGL3-P1848, pGL3-P1134. The fragments of the *Gnrh1* promoter (−520∼+23 bp, −356∼+28 bp, −249∼+28 bp), intron 1, intron 2 and intron 3 were amplified by PCR and subcloned into the pGL3-Basic vector or pGL3-Promoter vector to form the plasmids pGL3-P520, pGL3-P356, pGL3-P249 and pGL3-Int1–3. The details of the primers used in construction of plasmids were listed in Table S2.

The plasmids were transfected into cells using Lipofectamine 2000 (Invitrogen). In Otx2 overexpression experiment, the GT1–7 cells were plated on a 6-well plate (3×10^4^ cells/well) overnight. The cells in each well were transfected with 4 µg Otx2-myc-His/pcDNA3.1 or the corresponding empty vector as a control. In reporter gene assay, the GT1–7 cells were plated on a 96-well plate (1.5×10^4^ cells/well) overnight. The cells in each well were transfected with 0.4 µg of reporter plasmid or the corresponding empty vector (pGL3-Basic or pGL3-Promoter) as a control, along with 20 ng of pRL-SV40 as an internal control reporter. Twenty-four hours after transfection, HDACIs was added. The gene expression and the activities of firefly and renilla luciferases (Dual-Glo™ Luciferase Assay System, Promega) were detected 24 h after co-incubation. All experiments were performed in triplicate and repeated a minimum of three times.

### Western blot analysis

Total protein was extracted as described [43]. The nuclear protein was extracted by NE-PER® Nuclear and Cytoplasmic Extraction Reagents (Thermo). Equivalent amounts of proteins (25–40 µg) were separated by 15% SDS-PAGE and transferred onto a polyvinylidene fluoride (PVDF) membrane (Millipore). The membranes were sequentially incubated with antibodies overnight at 4°C. Immunoreactivity was detected with an enhanced chemiluminescence kit (Millipore). Histone 1 and β-Actin was used as loading controls.

### Chromatin immunoprecipitation (ChIP) assay

ChIP analysis was performed with Chromatin Immunoprecipitation (ChIP) Assay Kit (Millipore) according to manufacturer's instructions. Briefly, a total of 3×10^6^ GT1–7 cells in a 10-cm dish were treated with HDACIs for 24 h and then harvested. Then, the cells were lysed (200 µl SDS lysis buffer per 1×10^6^ cells) and sonicated (200 w for 5 sec with 10 sec interval, 100 cycles) to shear DNA to lengths between 200 and 1000 bp at ice box. Ten microliters of anti-Otx2 (Abcam) and anti-Acetyl-H3 (Cell signaling technology) was used to immunoprecipitate the interacting DNA fragments. The no-antibody immunoprecipitation was performed as the negative control. Finally, the DNA sample was used in a PCR reaction. The primers were listed in Table S3.

### Electrophoretic mobility shift assay (EMSA)

A total of 3×10^6^ GT1–7 cells in a 10-cm dish were treated with HDACIs for 24 h and then harvested. The nuclear protein was extracted by NE-PER® Nuclear and Cytoplasmic Extraction Reagents (Thermo). Biotin 5′ end-labeled and unlabeled probes containing the regions of *Gnrh1* promoter surrounding the confirmed Otx2 consensus sites (−268 to −239 bp and −330 to −301 bp) [19], [20] were chemosynthesized by Takara biotechnology CO. Binding reactions were prepared in EMSA binding buffer (Pierce) using 10 µg nuclear extract and a 20 fmol biotinylated probes, used on its own or together with either a cold competitor unlabeled probes (Cold probes, 4 pmol) or a specific supershift/blocking antibody (2 µg, Abcam). Reaction mixtures were incubated for 20 min at room temperature. Each sample was separated by gel eletrophoresis on a 5% nondenaturing polyacrylamide gel, and analyzed by chemiluminescence according to the instructions for LightShift® Chemiluminescent EMSA Kit (Pierce).

### Statistical analysis

All data were represented as mean ± SD with at least three independent experiments. The statistical significances among groups were analyzed by One-way ANOVA or *t*-test mostly comparing all values vs. control group. *P*<0.05 was considered to represent a significant difference.

## Supporting Information

Table S1 **Details of the primers used in the RT-PCR assay.**
(DOC)

Table S2 **Details of the primers used in construction of plasmids.**
(DOC)

Table S3 **Details of the primers used in ChIP assay.**
(DOC)
