# Supplementary material for: Histone Deacetylases Regulate Gonadotropin-Releasing Hormone I Gene Expression via Modulating Otx2-Driven Transcriptional Activity
Source: PLoS One. 2012 Jun 25;7(6):e39770. doi: 10.1371/journal.pone.0039770 (PMC3382570; doi:10.1371/journal.pone.0039770)
Supplement: Table S1 — Details of the primers used in the RT-PCR assay. (DOC) [file pone.0039770.s001.doc]

**Table S1 Details of the primers used in the RT-PCR assay.**

| **Name** | **5’-3’ sequence** | | **Products** |
| --- | --- | --- | --- |
|  | Forward primer | Reverse primer |  |
| *Hdac1* | GTGAACTACCCACTGCGAGAC | CACCTCCTCCCAGCATCA | 235 bp |
| *Hdac2* | ATTGGGCTGGAGGACTACAT | TCCCTCAAGTCTCCTGTTCC | 238 bp |
| *Hdac3* | GCCTCTGGCTTCTGCTAT | CGTAAGGGCACATTGAGA | 251 bp |
| *Hdac4* | TTCAGAACGGTGGTTATGC | AGCATTGGCATTGGGTCT | 309 bp |
| *Hdac5* | AGCTCAAGAATGGATTTGC | AGAAGTTCCCGTTGTCGTAG | 246 bp |
| *Hdac6* | CATGGTCAAGGAACACAGTT | AGCAATGTAGTCAGCGTCC | 198 bp |
| *Hdac7* | GAGGGCTTCAATGTCAACG | TCAACTGCTGCGTCATGTAC | 226 bp |
| *Hdac8* | CTCCAGAAAGTCAGCCAAGA | TTCCGTCGCAATCGTAAT | 269 bp |
| *Hdac9* | CAGAATCCTCGGTCAGTAG | GTTAGAAGCATTGAGTGGG | 251 bp |
| *Hdac10* | GGGCGTGTTGCGTTAGAG | CTGCTTAGACAGTGCGTGGA | 311 bp |
| *Gnrh1* | CACTGGTCCTATGGGTTGC | TTCTGCCTGGCTTCCTCT | 197 bp |
| *Otx2* | ACTTCGGGTATGGACTTGCT | CCTCACTTTGTTCTGACCTCC | 273 bp |
| *Grg4* | AACCCCCTTTGGGATCGTG | AGGTATTGCTGGCACTCGC | 196 bp |
| *GAPDH* | GTCCCTCACCCTCCCAAAG | GCTGCCTCAACACCTCAACCC | 233 bp |
| PCR conditions: 95°C for 1 min for denature, followed by 40 cycles of two-step PCR including melting for 10 s at 95°C，annealing for 20 s at 60°C. | | | |
