# Supplementary material for: Histone Deacetylases Regulate Gonadotropin-Releasing Hormone I Gene Expression via Modulating Otx2-Driven Transcriptional Activity
Source: PLoS One. 2012 Jun 25;7(6):e39770. doi: 10.1371/journal.pone.0039770 (PMC3382570; doi:10.1371/journal.pone.0039770)
Supplement: Table S2 — Details of the primers used in construction of plasmids. (DOC) [file pone.0039770.s002.doc]

**Table S2** Details of the primers used in construction of plasmids.

| **Name** |  | **5’-3’ sequence** | **Sites for restriction enzyme** |
| --- | --- | --- | --- |
| Otx2-myc-His/pcDNA3.1 | *Forward* | CGGGATCCAGAAATGATGTCTTATCTAAAGC | BamH I |
|  | *Reverse* | CCGCTCGAGCCAAAACCTGGAATTTCCA | Xhol |
| pGL3-P249 | *Forward* | AAGCTTAGAATGGTAGCTTCAG | Hind III |
|  | *Reverse* | AAGCTTGTACCTGTTTGGATGTG | Hind III |
| pGL3-P356 | *Forward* | GAAGATCTCAGTGTGTCTTTAACTTCAC | Bgl II |
|  | *Reverse* | CCGAAGCTTGTACCTGTTTGGATGTG | Hind III |
| pGL3-P520 | *Forward* | GGAAGATCTTCAGGCTGTCAGGATTG | Bgl II |
|  | *Reverse* | CCCAAGCTTCTGTTTGGATGTGAAAG | Hind III |
| pGL3-Int1 | *Forward* | GGTACCGTACAGTTCTTTGTTG | Kpn I |
|  | *Reverse* | CCCGGGTCCACTCTAAGGGAC | Smal |
| pGL3-Int2 | *Forward* | GAGCTCGTAAGTTTCCCAGCACT | Sac I |
|  | *Reverse* | CTCGAGCTGGAGATAAAGAATG | Xhol |
| pGL3-Int3 | *Forward* | ATCAAATGGCAGAACCCCA | Sac I |
|  | *Reverse* | AGTCCGTTCAACATTCATAAGC | Bgl II |
| PCR conditions: 95℃ for 5 min, followed by 25 cycles of three-step PCR including melting for 30 s at 95℃，annealing for 30 s at 56℃ and elongation for 1 min at 72℃. | | | |
