# Supplementary material for: Histone Deacetylases Regulate Gonadotropin-Releasing Hormone I Gene Expression via Modulating Otx2-Driven Transcriptional Activity
Source: PLoS One. 2012 Jun 25;7(6):e39770. doi: 10.1371/journal.pone.0039770 (PMC3382570; doi:10.1371/journal.pone.0039770)
Supplement: Table S3 — Details of the primers used in ChIP assay. (DOC) [file pone.0039770.s003.doc]

**Table S3** Details of the primers used in ChIP assay.

| **Name** | | **5’-3’ sequence** | **Products** |
| --- | --- | --- | --- |
| *Gnrh1* promoter (-1696~-1323bp) | *Forward* | CTTTGTGCCAGAGTAGTATGTT | 373 bp |
|  | *Reverse* | CTTTAGCAGATGCTGCCTCTAT |  |
| *Gnrh1* promoter (-1381~-1111bp) | *Forward* | GCATTTTTACAGCTTAGGTAGTGGA | 270 bp |
|  | *Reverse* | TCTGAAACTTTTGCTATCTTTACCC |  |
| *Gnrh1* promoter (-1196~-963bp) | *Forward* | GTGATACAAAGAGCTATTAACAGGC | 233 bp |
|  | *Reverse* | ATGGAACTCAAGACCTCTGGAAG |  |
| *Gnrh1* promoter (-1061~-751bp) | *Forward* | CCAGCCTCGTCTACATAGTTCC | 310 bp |
|  | *Reverse* | CTGTCCAGGAACCCACTTTGTAG |  |
| *Gnrh1* promoter (-845~-493bp) | *Forward* | TAAGCCAGGTGTGGTGGCA | 352 bp |
|  | *Reverse* | GGTTGCTCCCAATCCTGAC |  |
| *Gnrh1* promoter (-589~-410bp) | *Forward* | CACGAAGGTCAGAGGACAACT | 179 bp |
|  | *Reverse* | GAGAGCTTAGCATTTGGGTCTC |  |
| *Gnrh1* promoter (-483~-292bp) | *Forward* | GGAGCCATCTCACCAACACTAT | 191 bp |
|  | *Reverse* | AGCAGCCCAATGCAGAGTT |  |
| *Gnrh1* promoter (-382~-184bp) | *Forward* | GCATCACCTAACTTCCCTGTGAGT | 198 bp |
|  | *Reverse* | GCACGTTCTTAAACCTGGTCATT |  |
| *Gnrh1* promoter (-253~-89bp) | *Forward* | CCCTTAGAATGGTAGCTTCAGC | 164 bp |
|  | *Reverse* | GCAGCACAGCCCATAGTCTTA |  |
| *Gnrh1* promoter (-176~+24bp) | *Forward* | GACCAGCAGGTGTTGCAATTAC | 200 bp |
|  | *Reverse* | AGCTTCCGTTGGTAGGTTGAG |  |
| *PRL30* Control primer | *Forward* | TACCTTTGAGGCACGTTGTGTGAC | 158 bp |
|  | *Reverse* | ACCATCACCTCCATAATCCAGCCA |  |
| PCR conditions: 95°C for 5 min, followed by 35 or 40 cycles of three-step PCR including melting for 30 s at 95°C，annealing for 30 s at 58°C and elongation for 30 s at 72°C. | | | |
